# Meeting Report: Moving Upstream—Evaluating Adverse Upstream End Points for Improved Risk Assessment and Decision-Making

**DOI:** 10.1289/ehp.11516

**Published:** 2008-07-10

**Authors:** Tracey J. Woodruff, Lauren Zeise, Daniel A. Axelrad, Kathryn Z. Guyton, Sarah Janssen, Mark Miller, Gregory G. Miller, Jackie M. Schwartz, George Alexeeff, Henry Anderson, Linda Birnbaum, Frederic Bois, Vincent James Cogliano, Kevin Crofton, Susan Y. Euling, Paul M.D. Foster, Dori R. Germolec, Earl Gray, Dale B. Hattis, Amy D. Kyle, Robert W. Luebke, Michael I. Luster, Chris Portier, Deborah C. Rice, Gina Solomon, John Vandenberg, R. Thomas Zoeller

**Affiliations:** 1 Program on Reproductive Health and the Environment, Department of Obstetrics and Gynecology, University of California, San Francisco, San Francisco, California, USA; 2 Office of Environmental Health Hazard Assessment, California Environmental Protection Agency, Oakland, California, USA; 3 Office of Policy, Economics and Innovation, U.S. Environmental Protection Agency, Washington, DC, USA; 4 National Center for Environmental Assessment, U.S. Environmental Protection Agency, Washington, DC, USA; 5 Department of Medicine, University of California, San Francisco, San Francisco, California, USA; 6 National Resource Defense Council, San Francisco, California, USA; 7 Pediatric Environmental Health Specialty Unit, University of California, San Francisco, California, USA; 8 Wisconsin Division of Public Health, Madison, Wisconsin, USA; 9 National Health and Environmental Effects Research Laboratory, U.S. Environmental Protection Agency, Research Triangle Park, North Carolina, USA; 10 Institut National de l’Environnement Industriel et des Risques, Verneuil-en-Halatte, France; 11 International Agency for Research on Cancer, Lyon, France; 12 National Institute of Environmental Health Sciences, National Institutes of Health, Department of Health and Human Services, Research Triangle Park, North Carolina, USA; 13 George Perkins Marsh Institute, Clark University, Worcester, Massachusetts, USA; 14 School of Public Health, University of California, Berkeley, Berkeley, California; 15 National Institute for Occupational Safety and Health, Atlanta, Georgia, USA; 16 Environmental and Occupational Health Program, Maine Center for Disease Control and Prevention, Augusta, Maine, USA; 17 Department of Biology, University of Massachusetts, Amherst, Amherst, Massachusetts, USA

**Keywords:** adverse health effects, androgen antagonists, hazard identification, immunotoxicants, risk assessment, science policy, thyroid hormone, toxicologic assessments

## Abstract

**Background:**

Assessing adverse effects from environmental chemical exposure is integral to public health policies. Toxicology assays identifying early biological changes from chemical exposure are increasing our ability to evaluate links between early biological disturbances and subsequent overt downstream effects. A workshop was held to consider how the resulting data inform consideration of an “adverse effect” in the context of hazard identification and risk assessment.

**Objectives:**

Our objective here is to review what is known about the relationships between chemical exposure, early biological effects (upstream events), and later overt effects (downstream events) through three case studies (thyroid hormone disruption, antiandrogen effects, immune system disruption) and to consider how to evaluate hazard and risk when early biological effect data are available.

**Discussion:**

Each case study presents data on the toxicity pathways linking early biological perturbations with downstream overt effects. Case studies also emphasize several factors that can influence risk of overt disease as a result from early biological perturbations, including background chemical exposures, underlying individual biological processes, and disease susceptibility. Certain effects resulting from exposure during periods of sensitivity may be irreversible. A chemical can act through multiple modes of action, resulting in similar or different overt effects.

**Conclusions:**

For certain classes of early perturbations, sufficient information on the disease process is known, so hazard and quantitative risk assessment can proceed using information on upstream biological perturbations. Upstream data will support improved approaches for considering developmental stage, background exposures, disease status, and other factors important to assessing hazard and risk for the whole population.

To evaluate the potential of environmental chemicals to cause harm, to estimate the risks that chemical exposures pose to the population, and to identify opportunities for prevention and intervention, the type and extent of adverse effects associated with exposure to a chemical must be elucidated. To date, hazard and risk assessments have relied largely on data from traditional toxicologic studies, such as the 2-year, chronic toxicology and carcinogenesis studies or the two-generation reproductive toxicity assay. A primary goal of these studies is to identify whether chemical exposures cause overt disease outcomes, such as birth defects and neoplasia. These studies also provide data on biological events that precede these overt disease outcomes, often referred to as precursor effects. Adverse effects identified in existing hazard and risk assessments have often been the more overt diseases or defects, rather than events that occur earlier in the disease process.

Increasingly, toxicology assays are providing more information on how chemicals can interfere with cellular signaling or metabolism, disrupt hormone homeostasis, alter gene expression, or otherwise play a role early in disease processes. As scientific understanding of the mechanisms through which chemical exposures advance pathologic processes resulting in disease increases, so too does the opportunity for effective and efficient hazard identification and risk assessment. A necessary step in incorporating data on early biological perturbations is to consider how these early events relate to the concept of “adverse effects.” The U.S. Environmental Protection Agency (EPA) defines an adverse effect as “a biochemical change, functional impairment, or pathologic lesion that affects the performance of the whole organism, or reduces an organism’s ability to respond to an additional environmental challenge” (U.S. EPA 2007a) and, for example, considers such end points as alterations in circulating levels of sex hormones to be an adverse effect (U.S. EPA 1996). Identifying an adverse effect forms the basis for hazard identification and for defining the critical effect for quantitative risk assessment.

The evolution in toxicology testing from overt disease to elucidating toxicologic pathways has been recognized and endorsed by the National Academy of Sciences and federal agencies (Collins et al. 2008; National Research Council 2007). These organizations note that moving toward a focus on perturbations along the disease pathway should result in assays that can test more chemicals with reduced cost and time and fewer animals, and improve the scientific understanding of the relationships between exposures and health effects.

To move toward these goals, it is necessary to consider how data on early biological changes relate to the concept of an adverse effect and how these data might best be integrated into hazard and risk assessment. To this end, a workshop, Moving Upstream: A Workshop on Evaluating Adverse Upstream Endpoints for Improved Decision Making and Risk Assessment, was held 16–17 May 2007 in Berkeley, California.

## Workshop Summary

Three case studies were presented at the workshop that described available data on toxicologic pathways connecting chemical exposures to early upstream biological events and then to subsequent overt effects, which are considered “downstream”: *a*) thyroid hormone disruption and related toxicities; *b*) antiandrogen-mediated male reproductive effects; and *c*) upstream indicators of immunosuppression. To frame discussions in terms of implications for hazard identification and risk assessment processes, the case studies explored the following questions:

- What are the precursor effects or other early biological changes linked to the case study outcome?
- Is there sufficient evidence to associate the upstream events with the overt downstream effects?
- What related information would or does enhance our understanding of the relationship between the upstream event and downstream effects?
- Were the steps from the upstream event(s) to the overt downstream effect(s) identified? How does our understanding of these steps inform the use of the upstream event as a basis for risk assessment, particularly in situations when we only have data on upstream events?
- How does an understanding of variability in background biological status (e.g., susceptibility or sensitivity, genetic or otherwise) affect our interpretation of an upstream event?
- How might each end point be influenced by exposure to other chemicals?
- In considering an upstream biological end point that is measured as a continuous variable [e.g., thyroid-stimulating hormone (TSH)], what approach should be taken in deciding whether a certain degree of upstream change will lead to the overt downstream outcomes (e.g., neurodevelopmental change)? How should changes in upstream indicators within “normal” ranges be considered in evaluating potential for overt downstream outcomes in susceptible individuals and populations?
- Can the association between upstream events and downstream effects for chemicals considered in the case study be generalized to other chemicals that have the same upstream effects?
- What are the obstacles to the use of the upstream event in risk assessment (hazard identification; dose–response assessment)? How can they be overcome?

## Case Study Synopsis: Thyroid Hormone Disruption and Related Toxicities

### Thyroid hormone overview

The thyroid hormones (TH) thyroxine (T_4_) and triiodothyrine (T_3_) are essential to neurologic development, skeletal growth, and normal function of the pulmonary system, metabolism, and cardiovascular system. This case study emphasized the effects of thyroid disruption on neurologic development. Hypothyroidism is a condition of persistent deficits of TH and is diagnosed primarily by comparing an individual’s level of TH and TSH to population reference ranges. Untreated congenital (neonatal) hypothyroidism can have severe consequences on neurologic development. Children diagnosed with congenital hypothyroidism are treated with T_4_, and even small doses (e.g., 2 μg/kg/day) significantly improve later cognitive performance, demonstrating the sensitivity of the developing brain to TH insufficiency (Oerbeck et al. 2003; Selva et al. 2005).

Moderate maternal TH insufficiency during pregnancy can cause lasting developmental deficits in the child. Decrements in human maternal T_4_ during the early fetal period are associated with adverse outcomes such as reduced IQ scores, even for small T_4_ deficits that do not constitute maternal hypothyroidism (Haddow et al. 1999; Morreale de Escobar et al. 2000; Pop et al. 2003).

Levels of TH in the body are determined by a complex interplay of dynamic processes, including dietary intake of iodine (necessary for synthesis of TH); the transport of iodine into the thyroid; its synthesis and storage in the thyroid gland; its release and transport through circulation; the deiodination of T_4_ to T_3_ in peripheral tissues; and the degradation of TH by hepatic enzymes. TH homeostasis is regulated through a negative feedback loop involving the hypothalamus and pituitary. When TH levels decline, the hypothalamus signals the pituitary gland to release TSH, which stimulates the thyroid to increase TH. When levels rebound, the hypothalamus signals the pituitary to decrease TSH. Finally, TH action is mediated through TH receptors throughout the body.

In hazard identification and risk assessment, TH levels could serve as an upstream indicator of adverse effects on neurologic development. Table 1 provides an overview of classes, mechanisms of action, and effects for chemicals that disrupt TH homeostasis and action. For example, perchlorate inhibits the uptake of iodine, resulting in decreased synthesis of TH. Polychlorinated biphenyls (PCBs) and the pesticide acetochlor activate enzymes in the liver that increase excretion of TH, thus reducing circulating levels (Crofton 2008).

### Factors influencing effects of thyroid-disrupting chemicals

Susceptibility varies markedly with life stage. The fetus, infant, and child appear the most susceptible to neurologic disruption. The permanent effects of TH insufficiency on neurologic development were discussed above. Infants are likely less resistant to fluctuations in TH synthesis than adults. The adult thyroid stores several months’ supply of TH and the half-life of T_4_ in serum is 7–10 days (Greer et al. 2002; Vulsma et al. 1989). In contrast, the newborn thyroid stores a 1-day supply of TH and the half life of T_4_ in serum is 3 days (Vulsma et al. 1989). Therefore, effects on TH synthesis have more profound consequences for newborns than they do for adults.

### Recognizing subpopulations at risk is a key consideration in evaluating effects on TH levels or action as adverse

Hypothyroidism is prevalent in the U.S. population. Between 1999 and 2002, 7.3% of the U.S. population ≥ 12 years of age reported having thyroid disease or taking thyroid medication. Hypothyroidism is frequently undetected and therefore untreated. This condition often persists even in those who receive treatment. Among women receiving thyroid hormone replacement medication, 14% had TSH and T_4_ measurements indicating continued hypothyroidism. Pregnancy causes an increased demand on the thyroid gland, and hypothyroidism is twice as common during pregnancy (Aoki et al. 2007).

### The current method for assessing thyroid health may not be predictive of adverse downstream effects

**Figure 1:**
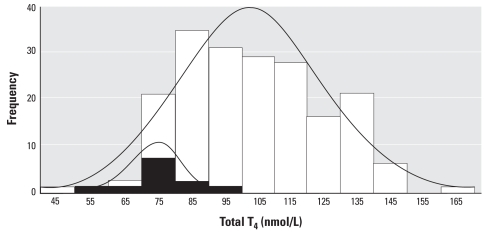
Distribution of 12 monthly measurements of total T_4_ in 15 healthy men (white bars) and one individual (black bars). The distribution in one individual is about half the width of the distribution in the group. Frequency represents number of measurements. Adapted from Andersen et al. (2002) with permission.

Thyroid health is evaluated primarily by comparing an individual’s level of TH and TSH to population reference ranges. However, there is a substantial variability among individuals in the level of TH that represents homeostasis, and the normal range of fluctuations for an individual is narrower than the normal range for a population (Figure 1). Consequently, a TH value within the population reference range is not necessarily normal or healthy for the individual (Andersen et al. 2002), and TH changes within the normal population range may therefore be associated with adverse effects for some individuals.

### Assessments considering thyroid-disrupting chemicals (TDCs) in isolation are likely to underestimate the potential disruption of TH levels or action by real-world chemical mixtures

Studies of human body burdens indicate simultaneous human exposure to multiple TDCs, including dioxins, PCBs, perchlorate, brominated flame retardants, bisphenol A, and several pesticides (Centers for Disease Control and Prevention 2008). A mixture of 18 TDCs (dioxins, dibenzofurans and PCBs) was tested at doses comparable to human exposure levels for effects on serum T_4_ in rats. Components of this mixture affect T_4_ through two different mechanisms of action: The dioxins, dibenzofurans, and dioxin-like PCBs in the mixture activate one set of liver enzymes, and the non-dioxin-like PCBs activate a separate set of liver enzymes. The mixture had a dose-additive effect on T_4_ at environmentally relevant doses and a 2- to 3-fold greater than dose-additive effect on T_4_ at higher doses (Crofton et al. 2005), demonstrating that exposures to chemicals acting on different pathways can have cumulative effects on an upstream marker. Assessments considering single TDCs in isolation are therefore likely to underestimate the potential disruption of TH by real-world exposure to chemical mixtures. It is appropriate to presume cumulative effects unless there is evidence to the contrary, and it is important for risk assessments to consider real-life exposure mixtures.

### Challenges for assessing risks of exposure to TDCs

Currently available mathematical models may not be able to accurately predict the effects of mixtures containing TDCs with multiple mechanisms of action. There are also numerous uncertainties in extrapolating thyroid data from laboratory animals to estimate effects in humans, including species differences in the expression or structure of specific functional proteins that may affect the toxicity of specific compounds.

### TH levels influence a spectrum of health outcomes and symptoms, in addition to neurologic development

**Lower** T_4_ and higher TSH levels are correlated with adverse effects on the pulmonary system (Mendelson and Boggaram 1991) and cardiovascular system, including increased blood pressure and less favorable blood lipid profiles (Asvold et al. 2007a, 2007b). A meta-analysis of 14 epidemiologic studies found an overall increase in risk of coronary heart disease of > 65% in those with subclinical hypothyroidism (elevated in TSH with normal T_4_) (Rodondi et al. 2006).

### Conclusions regarding thyroid data

Many environmental chemicals are capable of disrupting thyroid hormone levels, and many of these have the effect of decreasing circulating levels of T_4_. Compensatory mechanisms may not be sufficient to counteract the potential downstream consequences of these T_4_ decrements. First, findings in both animals and humans indicate that even small maternal T_4_ decrements within the normal population range during pregnancy can have adverse neurodevelopmental consequences, such as reduction in IQ, in the developing child. Second, fetuses and infants do not have stored thyroid hormone, and thus have limited capacity to respond to thyroid hormone decrements during critical stages of development. Third, there is a substantial prevalence of thyroid hormone insufficiency in the population of U.S. women, indicating that compensatory processes are already compromised for many individuals.

Therefore, for hazard identification purposes, workshop participants agreed that TDC exposures that would result in reduced T_4_ in a population should be considered an adverse effect. Further-upstream disruptions that result in lowered T_4_ levels, such as inhibition of iodine uptake, should similarly be considered adverse when their potential consequences include alteration of T_4_ levels. Because additivity or synergy of TDCs with different modes of action has been demonstrated, and background exposure to many TDCs is common, risk assessments should consider simultaneous exposures to multiple agents and account for these interactions.

Because pregnancy causes an increased demand on the thyroid gland, pregnant women are likely to have heightened sensitivity to thyroid toxicants, particularly if they have low dietary iodine or thyroid peroxidase antibodies. These sensitive subpopulations are likely to be sizable; for example, 38% of 126 pregnant women in the National Health and Nutrition Examination Survey (NHANES), 2001–2002, had low iodine intake (< 100 μg/L urinary iodine) (Caldwell et al. 2005). Because thyroid hormone levels affect cardiovascular risk factors, the potential impact of TDCs on the health of adults in the general population also is large. Therefore, effects on thyroid represent a risk to the general population and not just the developing organism.

## Case Study Synopsis: Antiandrogen-Mediated Male Reproductive Effects

### Overview of mammalian male reproductive development

Early in fetal development, male sexual differentiation is determined by expression of a Y chromosome gene, the sex-determining region Y (SRY). Appropriately timed SRY expression results in differentiation of Sertoli cells in the testes, initiating a cascade of events that result in development and differentiation of male reproductive structures and regression of female structures. The interstitial cells of the testes, Leydig cells, produce two important hormones for male reproductive development: the steroid sex hormone testosterone and the peptide hormone insulin-like-3 (INSL-3). Testosterone is necessary for differentiation of the Wolffian duct into the epididymis and secondary sex organs such as the seminal vesicles. Both testosterone and INSL-3 are necessary for testicular descent in mammals: Testosterone mediates transabdominal descent of the testes (Klonisch et al. 2004), whereas INSL-3 is necessary for gubernacular ligament development. Contraction of the gubernaculums causes the inguinoscrotal descent of the testes into the scrotum (Wilson et al. 2004). Disruption of testosterone and/or INSL-3 production or action can result in cryptorchidism (undescended testes), one of the most common birth defects in humans.

### Exposure to antiandrogens during fetal life

During fetal life, there is a transient peak in testosterone levels necessary for proper development of male reproductive tissues. Disruption in androgen action during this critical time window results in a number of abnormalities that consistently develop in laboratory animals (e.g., rats and rabbits), including retained female structures, such as nipples in male rodents, and malformations of male reproductive structures, such as hypospadias (an abnormal location of the urethral opening) and cryptorchidism.

Multiple modes of action can disrupt androgen activity, including a decrease in testosterone production or interference with androgen receptor binding. Each of these modes of action has been well described in the literature for two different classes of chemicals with antiandrogenic effects.

First, carboximide pesticides such as linuron, vinclozolin, and procymidone are classic antiandrogens in that they bind antagonistically to the androgen receptor (AR), interfering with endogenous testosterone and dihydrotestosterone binding to the AR, and thus decreasing the expression of androgen-dependent genes. In fetal rat studies, exposure to these chemicals has been consistently associated with retained nipples, decreased sperm counts, decreased anogenital distance, hypospadias, and decreased size or agenesis of the accessory sex glands (Gray et al. 2006). The severity of effects increases with the dose.

Second, there are certain phthalates—industrial chemicals with widespread exposure in the general population—that do not bind to the AR but do interfere with androgen activity by inhibiting testosterone synthesis. Specifically, phthalates with four to six carbon side-chains interfere with testosterone production by inhibiting cholesterol uptake into the mitochondria by steroidogenic acute regulatory protein and by inhibiting some, but not all, of the enzymes in the steroidogenic pathway (Barlow et al. 2003). These phthalates have been most consistently shown to reduce testosterone production in animal research to date, though other phthalates may be important (Gray et al. 2000; Liu et al. 2005).

Rat studies have demonstrated that a phthalate-induced decrease in testosterone results in a syndrome of anti-androgenic reproductive abnormalities, referred to as the “phthalate syndrome,” which is characterized by malformations of the epididymis, vas deferens, seminal vesicles, and prostate; hypospadias, cryptorchidism, and testicular injury; permanent changes (feminization) in the retention of nipples and areolae (sexually dimorphic structures in rodents); and demasculinization of the growth of the perineum, resulting in a reduced anogenital distance (Mylchreest et al. 2000). As with other antiandrogenic chemicals, the severity of effects increases with dose.

The carboximide pesticides and four to six side-chain carbon phthalates have different modes of action resulting in the same spectrum of adverse outcomes in male reproductive development. In the developing male reproductive tissues, disruption of either of these two parts of the androgen action pathway converges into a final common pathway: a decrease in androgen-dependent gene expression.

The spectrum of effects seen in phthalate syndrome parallels a spectrum of human diseases called “testicular dysgenesis syndrome”: infertility, cryptorchidism, hypospadias, and testicular cancer. Several epidemiologic studies have shown associations between certain phthalate metabolites and male developmental reproductive outcomes, including shortened anogenital distance in male infants prenatally exposed to phthalates (Swan et al. 2005), changes in reproductive hormone levels in male infants exposed to phthalates in breast milk (Main et al. 2006), and poor semen quality in adult males (Hauser et al. 2006). The exposure levels in most subjects in these studies were comparable to the general population exposures.

### Antiandrogen effects as adverse end points in hazard identification and risk assessment

An antiandrogenic agent, therefore—one that reduces androgen action by any mode of action (e.g., binding to the androgen receptor, or via interference with androgen synthesis)—can be predictably associated with a series of adverse end points in animals and likely also in humans. Several additional scientific findings need also be considered when considering antiandrogen effects as upstream adverse end points.

#### Specific periods during development are uniquely susceptible to perturbations

Experiments in rats have demonstrated that both the dose and the timing of exposure are important for development of phthalate syndrome. Gestation days 12–19 have been identified as a sensitive period in rats, during which exposure to dibutyl phthalate induces phthalate syndrome, with some irreversible effects (Carruthers and Foster 2005).

Hydrolytic phthalate monoesters are the bioactive metabolites responsible for phthalate toxicity. Excretion of phthalate monoesters in urine is enhanced by glucuronide conjugation. Experiments in rats have shown the glucuronidation pathway is immature and inefficient during the period of susceptibility for phthalate syndrome. This relatively low level of glucuronidation compared to adults could render the fetus much more susceptible to the biologically active phthalate metabolites during a critical period of sexual development. Exposure to rats during this period of gestation has demonstrated the proportion of free phthalate monoesters relative to glucuronidated phthalate monoesters in amniotic fluid is much higher than levels in the urine of the pregnant dam (Calafat et al. 2006).

#### Exposure to mixtures of AR antagonists and androgen synthesis disruptors

More than 95% of the population from 6 to > 65 years of age are exposed to at least five phthalates on a regular basis (Silva et al. 2004). Recent studies show that exposure to mixtures of chemicals that interfere with androgen action results in dose-additive effects. Rats exposed to a mixture of AR antagonists, vinclozolin, procymidone, and flutamide, all acting through the same mode of action, at doses that would not have caused hypospadias alone, resulted in > 50% of the animals having hypospadias (Christiansen et al. 2008). Rider et al. (2008) found that pre-natal exposure to a mixture of seven phthalates and pesticides with differing modes of action (i.e., AR antagonist or inhibition of androgen synthesis) produced cumulative, dose-additive outcomes in the androgen-dependent tissues.

#### Exposures to phthalates can result in adverse effects from modes of action other than disrupting testosterone synthesis

In addition to inhibiting the production of testosterone, phthalates also interfere with production of INSL-3, which is necessary for gubernaculum development (Wilson et al. 2004) and subsequent descent of the testis into the scrotum. Unique to phthalate exposure is an absence of the gubernacular ligament in males exposed *in utero.* Thus, phthalates can act through two different modes of action to cause cryptorchidism.

### Conclusion regarding antiandrogen data

The prenatal exposure of males to antiandrogenic chemicals illustrates several important points when considering upstream indicators of adverse effects: First, it is necessary for exposure to occur during the critical window, the period of reproductive organ development, in order for certain developmental effects to be observed. Second, perturbations early in the development of the male reproductive system predictably result in a wide array of adverse outcomes that are permanent and irreversible. Third, exposures to different chemicals with different modes of action can result in the same outcomes due to a deficiency in androgen-mediated gene expression. Finally, exposure to one chemical can have multiple modes of action with effects on the same end point. In summary, exposures to antiandrogens can cause adverse effects in upstream indicators, including a reduction in fetal testosterone levels or androgen receptor binding, which can increase the risk of a constellation of downstream effects including cryptorchidism, hypospadias, and, later in life, infertility.

## Case Study Synopsis: Immune Function, Immunotoxicity, and Resistance to Infection and Neoplasia: The Downstream Impacts of Unintended Immunosuppression

### Immune system overview

The immune system consists of a complex system of tissues, cells, and soluble mediators that protects the body against foreign substances, including infectious agents and some types of tumor cells. Immune cells are located throughout the body, either in discrete organs, such as the spleen, thymus, and lymph nodes, or in diffuse accumulations of lymphoid and myeloid cells, as are found in association with the skin, lungs, and gastrointestinal tract. Destruction of infectious agents relies on both innate and adaptive immune responses. Innate responses are rapid, do not require clonal expansion, and are stimulated by recognition of pathogen-associated molecules by macrophages, natural killer cells, and granulocytes. Adaptive, or acquired antigen-specific responses, rely on antigen recognition and subsequent events that culminate in cell proliferation and maturation, mediator production, and generation of long-lived memory cells that respond rapidly on subsequent exposures to the same or closely related antigens. The acquired immune response is mediated by two types of lymphocytes, T cells and B cells. T cells are a source of soluble mediators known as cytokines that stimulate other immune system cells, including B cells, and act as effector cells with cytotoxic activity. B cells mature into plasma cells, which serve to produce immunoglobulins (Ig), or antibodies, of various subclasses, including IgM, IgG, IgE, IgA, and IgD. Each of these antibody subclasses serves a unique function in the immune response.

### Factors influencing immune function

Numerous factors influence the outcome of an encounter with an infectious agent. Innate defenses, critical in the early phase of resistance, may be overcome by large numbers of infectious agents. The virulence of the pathogen may also prevent effective innate and acquired responses from eliminating microorganisms before infection ensues. Host attributes such as age, sex, genotype, lifestyle, and disease status all influence immunocompetence, and each may influence the incidence or severity of infections.

Newborns are not immunologically mature at birth and are thus at increased risk of infection. Protective antibodies are transferred from mother to fetus across the placenta and to the newborn in breast milk, although passive protection decreases rapidly as these proteins are catabolized. Average IgM and IgG antibody levels do not reach 50% of adult levels until 7–12 months, and IgA reaches adult levels by 3–5 years. A preponderance of naive T cells (90% vs. 50% in adults) contributes to reduced cell-mediated immunity in the young. Immunocompetence also declines with advanced age; however, few studies have evaluated the effects of immunotoxicants in older laboratory animals and humans.

Studies in laboratory animals and humans have established that extrinsic factors, including environmental chemicals, drugs, physical agents, and psychological factors may influence the course of infection. Furthermore, unintended immunosuppression resulting from exposure to environmental chemicals alone or in combination with other intrinsic and extrinsic factors can shift the distribution of normal immune response, resulting in an increase in individuals classified as “immunosuppressed.” Immunosuppressed individuals might express alterations of immune function such as reduced antibody production, decreased immune cell counts, or ineffective cell signaling. Recent data also suggest that, in addition to suppression, developmental exposure (i.e., exposure from birth through puberty) to certain chemicals may shift the pattern of cytokine production, leading to a greater incidence of allergy and asthma (Luebke et al. 2006).

### Effects on the immune system during periods of susceptibility

In a review of select developmental immunotoxicology literature, Luebke et al. (2006) concluded that exposure to environmental chemicals during key developmental periods can affect immune function in laboratory animals. Developmental exposure suppresses T-cell function through adolescence in mice and throughout adulthood in rats. Gestational/neonatal exposure to diazepam (DZP) and diethylstilbestrol (DES) in rodents can cause long-lasting (up to lifetime) effects on the immune system, including suppression of IgM and IgG antibody responses and nonspecific lymphocyte proliferation (DES) at doses that cause only short-term immunotoxicity in adults. Developmental exposure to DZP, lead, or tributyltin oxide caused immunotoxicity at lower doses in young than in mature animals, and developmental effects were persistent. Clinical experience indicates that adult immune function typically recovers soon after therapeutic immunosuppressive treatment ends. However, the developmental data suggest that screening chemicals exclusively in adult animals may fail to detect persistent effects or those effects that occur at lower doses (Luebke et al. 1999).

Immune function also declines with age, as do the normal physiologic processes that limit microbial invasion. Although data are limited, this suggests that relatively small changes in immune function resulting from chemical exposure may have more severe consequences if combined with normal immunosenescence.

Several epidemiologic studies have described associations between early-life chemical exposure, altered immune end points, and frequency of infections. Increased incidence of otitis media and respiratory infections were reported in children exposed to PCBs (Dallaire et al. 2006; Dewailly et al. 2000; Nakanishi et al. 1985; Weisglas-Kuperus et al. 2000; Yu et al. 1998). For example, at 3 months of age, multiple upstream indicators of impaired immune function were detected in breast-fed infants with higher PCB exposure levels, including reduced numbers of white blood cells and lymphocytes, and lower serum IgA levels at 7 and 12 months of age (Dewailly et al. 2000). These studies also reported changes in immune system biomarkers, such as changes in blood cell counts and T-cell subsets or decreased serum Ig levels with increasing PCB exposures (Karmaus et al. 2001; Weisglas-Kuperus et al. 2000). Antibody responses to tetanus toxoid vaccine were significantly decreased after early postnatal PCB exposure (Heilmann et al. 2006).

Individuals with very severe forms of immunosuppression, as occur in primary immunodeficiency diseases and AIDS, frequently develop opportunistic infections. Xenobiotic agents are likely to cause considerably more subtle immunologic effects. The interaction among host genetics, pathogen virulence, and pathogen dose plays a role in determining the frequency and severity of illness. For individuals exhibiting mild immunosuppression, as may be associated with xenobiotic chemical exposure, the infectious dose of a given pathogen may be lower than that which would cause disease in an individual whose immune system is functioning optimally. Limited studies in populations with mild forms of immunosuppression (e.g., under psychological stress or administered immunosuppressive therapies) have provided qualitative evidence that moderate changes in immune function can also lead to an increased incidence of infectious or neoplastic diseases (Cohen et al. 1991; Kiecolt-Glaser et al. 2002).

### Conclusions regarding immunosuppression data

In summary, exposures to environmental contaminants have the potential to affect upstream immune indicators, including antibody synthesis, T-cell function, and other measures of immunocompetence, which can result in compromised downstream resistance to infection. However, the impact of background levels of xenobiotics on the burden of disease has not been clearly established. If a positive correlation between increasing exposure and disease is assumed, then even small changes in immune function will represent an increased risk for developing disease.

## Common Themes

The case studies demonstrate that certain toxicity pathways are understood well enough that screening assays to identify early biological changes could be used for hazard identification and risk assessment. For example, if a chemical is found to suppress thyroid function, the associated data could be used to assess risk for developmental neurotoxicity, because the latter effect can be predicted on the basis of the mechanism of action alone. Similarly, when a chemical is found to be antiandrogenic, the current science would support using the data to assess increased risk of developmental effects. Data from whole-animal tests of the downstream outcomes would not be required for risk assessment of these outcomes. The findings of this workshop support the recent National Academy of Sciences recommendation that toxicity testing move toward the direct assessment of upstream events along toxicity pathways and gradually move away from the current whole-animal end point–based assays (National Research Council 2007).

The workshop also explored some of the challenges that arise when using upstream events to assess the adverse effects of chemical exposures during hazard identification or risk assessment. Several common themes emerged from the case study discussions. Some of these themes are not unique to the use of upstream indicators as the basis of hazard identification and risk assessment. Rather, they underscore issues that should be addressed in existing hazard identification and risk assessment practices.

### Chemical and biological background

**Figure 2:**
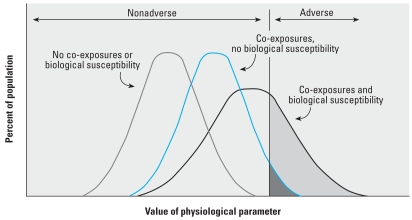
Distribution of a typical physiological parameter within the population and how that may vary depending on the influence of chemical and biologic background.

Each of the case studies illustrated the importance of considering preexisting or continuous exposure to environmental chemicals as well as preexisting biological or disease susceptibilities that contribute independently to risk of overt disease. Preexisting exposures or biological vulnerabilities can increase the baseline risk of the population or enhance already initiated disease processes (Figure 2). Consequently, slight perturbations in upstream biological indicators are more likely to increase risk of subsequent downstream events given an already more activated state among segments of the population.

#### Chemical background and exposure to mixtures

NHANES data indicate that the entire U.S. population has measurable levels of multiple environmental contaminants in their bodies (Centers for Disease Control and Prevention 2008). Other studies of exposure pathways show the population is in constant contact with xenobiotics in air, food, water, and consumer products (Environmental Working Group 2005, 2007; National Library of Medicine 2008; U.S. EPA 2007b; U.S. Food and Drug Administration 2007). U.S. EPA guidelines for mixtures risk assessment recommend default assumptions of dose additivity for mixtures of chemicals with similar toxicologic activity and response additivity for mixtures of chemicals that act independently (U.S. EPA 2000a). The case studies showed that these preexisting and concurrent exposures can increase the effect a given chemical exposure has on disease risk, consistent with the dose-additivity default. For example, separate studies of antiandrogenic chemicals and thyroid-disrupting chemicals found that mixtures of chemicals acting on common systems via different modes of action had dose-additive effects. Although a less-than-additive response [referred to in the U.S. EPA mixtures guidelines as “antagonism” (U.S. EPA 2000a)], or a greater than additive response (“synergism”) are possibilities, the case studies support application of dose additivity as a default, as recommended in the guidelines. Considering the influence of multiple chemical exposures on upstream events can facilitate a more realistic characterization of the risks of downstream effects when assessing a single chemical that is an additional increment to the background exposure mixture.

#### Biological background

Background health status, as influenced by age, preexisting disease, genetics, and other factors, can influence the effect of chemical exposure on upstream and downstream adverse end points. For example, the implications of a chemical’s interference with iodine uptake into the thyroid are substantially greater for an infant than for an adult because of differences in thyroid hormone storage and circulating half life. Likewise, the implications are greater for the 38% of pregnant women in the United States who are deficient in iodine (Aoki et al. 2007) and who simultaneously have greater thyroid hormone needs to support the neurologic development of a fetus. Individuals with pre-existing immune suppression, such as organ transplant patients, those who are HIV positive, and those at early or late life stages, might experience disproportionately higher disease risk due to chemical exposure.

### Periods of susceptibility

Exposure to environmental chemicals during periods of susceptibility can pose a unique risk of subsequent downstream effects, both in the short and long term, as well as diminished capacity for recovery from decrements to physiological systems. For example, exposure to thyroid-disrupting chemicals such as perchlorate during the fetal and early child development can produce adverse effects on neurologic development by decreasing TH levels; the same mild perturbations in TH levels would not present an adverse challenge to a healthy adult. Similarly, to impair male reproductive development in rodents, exposure to phthalates must occur during gestation days 12–19. When exposure occurs during periods of developmental susceptibility, the dose required to produce an effect will typically be lower than the dose required to produce effects in fully developed, healthy adults.

### Multiple or complex modes of action

Exposure to a chemical can influence disease processes through multiple modes of action and also increase the risk of more than one downstream, overt effect. Hence, measurements that focus on a particular pathway or individual downstream event may provide an incomplete picture of the range of effects produced by the chemical. Phthalates work through multiple pathways to decrease androgen production but also interfere with the production of INSL-3. The resulting deficits in these hormones during male fetal development can increase the risk of several different downstream effects, both manifested early in life (e.g., cryptorchidism) and later in life (e.g., effects on sperm). Data interpretations therefore should consider the possibility of a complex set of modes of action that produce adverse effects on the whole system. Focusing on one single aspect of these complex modes of action can result in inadequate characterization of system response.

### Continuous versus discrete events

Overt downstream effects, such as cancer, birth defects, and infectious illnesses are typically discrete events. In contrast, upstream indicators of adverse effects, such as changes in hormone levels or cellular markers of immune function, are often measured on a continuous scale. Risk assessment practices recognize differences in frequency of discrete events as an indication of an adverse effect. Characterization of changes in continuous outcomes in risk assessment requires further consideration.

One key issue is assessing the level of change constituting adversity. Should any chemical-induced increase or decrease be considered adverse? Should there be a focus on particular cut points in the distribution of population or individual baseline levels? These questions should be considered in the context of the issues of background exposures and age-related susceptibilities noted above. One possibility is to consider that deviations from expected baseline (e.g., for a given individual, with all its characteristics, susceptibilities, age, sex, physiologic state) will increase the probability (risk) of downstream adverse effects. For example, changes in biological function during susceptible periods, such as during development, can increase risk of subsequent downstream events compared to biological function changes of the same magnitude at other life stages. Whether the perturbation is reversible and whether it is possible to use population reference ranges to detect adversity are two key questions that may arise in the process of considering continuous upstream indicators of adverse effects.

### Reversibility

Biological perturbations resulting from chemical exposure are often evaluated in regard to whether they are reversible. For example, exposure to chemicals can result in immune system suppression and increased opportunity for infections. A suppressed immune system may recover once chemical exposure is stopped, and the risk of infectious disease returns to original levels. However, for assessments of a constant chronic or lifetime exposure, recovery from shorter duration exposures should not be considered when evaluating whether the observed response is adverse. Whether a perturbation is reversible can depend on intrinsic factors, such as age or disease status, and extrinsic factors, such as pre-existing or co-exposures to other contaminants or the timing of exposure. For example, gestational and neonatal exposure to DZP and DES in rodents can cause long-lasting effects on immune function; in contrast, adult rodents exposed to the same dose experience reversible effects (Luebke et al. 2006).

### Population variability and defining the normal range

Basing the definition of “normal” function for a biologic parameter on population reference ranges may not reflect an individual’s normal range or variability and may fail to detect an adverse effect. For example, an individual may experience an adverse decline in thyroid hormone but still remain within the normal population reference range (Figure 1). For situations in which the interindividual variability in a continuous upstream indicator is high, alternative methods to detect adverse changes in the biologic parameter will need to be developed.

## Conclusions and Recommendations

Workshop participants concluded that for the toxicity pathways represented in these case studies, it may be feasible to move toward using direct evaluation of upstream mechanistic indicators as the basis for risk assessment and decision-making.

The case studies illustrate the complexities of the biological changes influenced by exposures to environmental contaminants. Capturing and translating the complexities of the science is an ongoing challenge in the regulatory and policy arena. While science pursues new areas of inquiry, decision making requires timely answers to questions about risks and hazards to public health in order to mitigate future or current potential harm. The regulatory context also requires a different sufficiency of evidence whereby regulatory decisions can be made based on evidence that a chemical is likely to cause a particular outcome. The value of a parallel-tracks approach was noted, in which scientific investigation continues in one track while development of appropriate principles and practices for assessing the emerging science for timely public health–based decision-making continues in another.

### Identifying classes of upstream events

The case studies of thyroid perturbation, anti-androgen activity, and certain types of immune changes each represent a class of perturbations whose links to downstream events were determined by workshop participants to be sufficiently characterized by the data to move toward considering issues of implementation. A suggested next step is to evaluate chemicals for their ability to cause the upstream perturbation identified in the case studies. For these chemicals, hazard and risk assessment could be initiated with data on the relationship between exposures and early perturbations. Fewer data are needed for subsequent downstream events, given what is already known on risks linking the pathway from early to late events, and with the presumption that downstream consequences are likely to occur when the upstream perturbation is observed.

Participants noted other types of early biological perturbations that were not discussed in the case studies but that are also likely sufficiently characterized as to be defined as a class. Early biological perturbations that appeared to be sufficiently characterized include certain high affinity and persistent interactions with the Ah receptor, changes in hormonal responses [which are identified in reproductive toxicity risk assessment guidelines as an adverse event (U.S. EPA 1996)], and cholinesterase inhibition [which has been identified as an indicator of adverse effects (U.S. EPA 2000b)]. It would also be beneficial to expand the list to include biological perturbations that appear common to many environmental chemicals, such as effects on birth weight or liver function.

Participants emphasized that additional information on upstream indicators does not necessarily reduce uncertainty, although it does add more information about the exposure–disease continuum and increases the predictive power of risk assessment and can demonstrate greater variability across study subjects than previously recognized. Hence, using upstream indicators does not necessarily mean reducing applicable uncertainty or adjustment factors.

### Next steps

The case studies suggest chemical exposure–induced changes in upstream biological markers have utility for hazard identification and risk assessment. The approaches to using these upstream indicators should consider factors such as biological background, chemical background (and the potential for dose additivity or synergism), periods of susceptibility, and multiple and complex modes of action that can increase the risk of subsequent downstream events. The case studies also illustrated the need to better integrate the nonzero baseline concept into hazard and risk assessment—in other words, background exposures and processes can confer a greater potential for effects than when exposure to a chemical is considered in isolation. An important implication of this concept is that the current framework for addressing risks from noncancer health effects no longer fits with what we know about the science and needs of the decision-maker for current understanding of potential risk. Disease status, susceptibility, and chemical background are critical to consider when assessing the implications of exposure, as they can put a portion of the population in the dose range where small incremental exposures can increase risk of downstream events. This suggests that the assumption of a threshold dose level—below which no deleterious effects occur (U.S. EPA 2002)—may not apply, and incorporating concepts traditionally used for nonthreshold events should be considered.

## Figures and Tables

**Table 1:** Classes, mechanisms of action, and effects of thyroid-disrupting chemicals on thyroid hormone homeostasis.

Class	Mechanism	Effects on thyroid hormones	Chemicals
Iodine transport	Inhibit uptake of iodine	Decreased thyroidal synthesis of T_3_ and T_4_	Perchlorate, chlorate, bromate, nitrates, thiocyanate
Synthesis inhibitors	Inhibition of thyroperoxidase	Decreased thyroidal synthesis of T_3_ and T_4_	Methimazole, propylthiourea, amitrole, mancozeb, soy isoflavones, benzophenone 2, 1-methyl-3-propyl-imidazole-2-thione
Transport disruption	Altered binding to serum transport proteins	Unknown	Hydroxylated PCBs, EMD 49209; pentachlorophenol
Enhanced hepatic catabolism	Up-regulation of glucuronylsyltransferases or sulfotransferases (via CAR/PXR or AhR)	Increased biliary elimination of T_3_ and T_4_	Acetochlor, phenobarbital, 3-methylcholanthrene, PCBs, 1-methyl-3-propyl-imidazole-2-thione
Enhanced cellular transport	Up-regulation of OATPs or MCT transporters via CAR/PXR or AhR	Increased biliary elimination of T_3_ and T_4_	TCPOBOP, pregnenolone-16α-carbonitrile, TCDD, rifampicin, phenobarbital, oltipraz
Sulfotransferases	Inhibition of sulfotransferases	Decrease sulfation of THs	Hydroxlyated PCBs, triclosan, pentachlorophenol
Deiodinases	Inhibition or upregulation of deiodinases	Decreased peripheral synthesis of T_3_	FD&C Red dye no. 3, propylthiouracil, PCBs, octyl-methoxycinnamate
TR agonists and antagonists	Direct or indirect alterations in TR–TRE binding	Altered activation of TH-dependent gene transcription	Tetrabromobisphenol A, bisphenol A, hydroxylated PCBs

Abbreviations: AhR, aryl hydrocarbon receptor; CAR: constitutive androstane receptor; FD&C, Federal Food, Drug, and Cosmetic Act (1938); MCT, monocarboxylate transporter; OATPs, organic anion-transporting polypeptides; PXR, pregnane X receptor; TCDD, 2,3,7,8-tetrachlorodibenzo-*p*-dioxin; TCPOBOP, 1,4-*bis*[2-(3,5-dichloropyridyloxy)]benzene; TH, thyroid hormone; TR, thyroid receptor; TRE, thyroid hormone response elements. Data from Crofton (2008).
